# When passion is not enough: the waning of the infectious disease workforce

**DOI:** 10.1128/msphere.00902-24

**Published:** 2025-05-05

**Authors:** Jennifer A. Leeds

**Affiliations:** 1 Independent Consultant, Walnut Creek, California, USA; University of Michigan, Ann Arbor, Michigan, USA

**Keywords:** infectious diseases, talent, incentives

## Abstract

Scientists and physicians who pursue careers in infectious diseases are often passionate about understanding microbial pathogenesis and anti-microbial resistance. They are driven by the impact that their work has on improving public health and reducing human suffering through the discovery, development, and delivery of technologies that enable prevention and radical cure of debilitating and deadly diseases. Yet, despite their passion, there is a shrinking talent pool in antibiotic research and development (R&D) (https://www.amrindustryalliance.org/mediaroom/leaving-the-lab-tracking-the-decline-in-amr-rd-professionals/) as well as in the clinical practice specialty of infectious diseases as recognized by the Infectious Diseases Society of America (https://www.idsociety.org/news--publications-new/articles/2024/idsa-and-pids-statement-on-2024-id-fellowship-match/). This talent drain has wide repercussions beyond its impact on R&D and clinical practice and needs to be addressed from many angles to attract and retain new talent, to increase investment in and output from antibiotic R&D, and to mitigate the rising risk of the inability to deliver safe and efficacious solutions to patients in need.

## PERSPECTIVE

I have been destined to be a microbiologist since I was 11 years old. My mother worked in a urology practice and would come home with scary stories about urinary tract infections and sexually transmitted diseases. I was blown away by the power that a tiny single-celled life form could wield over a human being. I was even more awed (and terrified!) when those wee animalcules were unable to be successfully defeated by available therapeutics. You have to respect entire genera of one-celled creatures without brains and hearts and arms and legs that are nevertheless capable of outcompeting a complex human immune system and modern medicine. I knew, then, that I had to study microbiology and, more specifically, microbial pathogenesis and antimicrobial resistance (AMR).

In college, microbiology was largely focused on fermentation and microbial physiology. There were only six of us in the major in my graduating class, and microbial pathogenesis was taught by one very dedicated faculty member who left the university midway through my tenure. My craving for more knowledge was largely met by reading entries in centuries-old lab notebooks, in the bowels of the veterinary school library!

In graduate school, I was surrounded by experts in microbial pathogenesis who encouraged my passion to advance my career in the discipline. Nevertheless, many esteemed scientists coached me to abandon the area and move into the burgeoning field of eukaryotic “signal transduction.” After all, they said, “No one dies from an *E. coli* infection!”

Having attempted to get excited about signal transduction and failing miserably, I remained true to my deep love for bacterial genetics and enjoyed a fruitful postdoctoral experience followed by a first foray into biotechnology. Neither, however, was focused on pathogenesis and antibiotic resistance.

Not long after leaving academia, I heard about this new company called Novartis and learned that they were building an infectious diseases (ID) research and development (R&D) team. I had re-discovered my calling! I spent the next 16 years leading anti-bacterial research teams and ultimately a whole organization of drug discoverers in a shared passionate pursuit to understand deeply what drove anti-microbial resistance and to discover and develop novel therapeutics to tackle this growing challenge. And yes, people **do** die from *Escherichia coli* infections!

By 2017, our group had achieved the peak of our performance, with a strong and diverse portfolio and with over 100 fantastic colleagues engaged in anti-bacterial R&D. We moved novel antibiotics addressing unmet medical needs into clinical development, had impactful drug discovery efforts under way, and were heavily invested in understanding “bacterial pharmacokinetics” with an eye toward overcoming the challenges of the Gram-negative permeability and efflux barriers. We were working on new commercial models for antibiotics and were lobbying for incentives to keep big pharma in the game.

Then, it all came to a grinding halt in mid-2018 when the company decided to exit the anti-bacterial and anti-viral business (https://www.genengnews.com/insights/as-novartis-exits-who-will-make-new-antibiotics/). It takes time to build a well-functioning anti-microbial discovery team and portfolio. Antibiotic discovery is scientifically challenging, and yet the potential for commercial success with newly launched antibiotics, which are typically second- or third-line products largely based upon policy around reimbursement, is limited in comparison with new products from other therapeutic areas where there can be considerably higher returns on investment.

In a matter of months, we wound down R&D efforts, cleaned out labs, drafted manuscripts, and put more than a dozen years of data and bacterial strain collections into the public domain, hosted career fairs, and packaged programs for out-licensing. Forty dedicated anti-bacterial discovery scientists, along with an equal number of anti-viral discovery colleagues, dozens of medicinal chemists, pharmacologists, physicians, preclinical and clinical sciences colleagues, and the entire ID leadership team, were seeking new opportunities, and we were not alone. So many of our peers had recently experienced or were about to experience similarly dramatic exits from the field about which they were so passionate and invested. Of the roughly 100 colleagues working on our anti-bacterial R&D efforts, only three still remain in anti-bacterial R&D full time (all in a single organization). The rest of us went into different therapeutic/technology areas, and a few of us have since retired from full-time work in biopharma.

I transitioned, within Novartis, to business development and was fortunate to learn about what motivates scientists to build companies and to pursue drug R&D across a host of therapeutic areas; what motivates investors to bet on talent, technology, and assets; and what drives technology licensing and mergers and acquisitions. Over the next 6 years, I was able to more broadly experience and participate in the biopharma, academic, government, and non-government organization therapeutic and diagnostic arena, which helped me to see the challenges of the anti-bacterial business and the ensuing talent drain through a different lens.

At the time that I was facing my own decision of whether to stay in or leave anti-bacterial R&D, I weighed the pros and cons heavily. On the plus side, bacterial pathogenesis and AMR are my passion and my first love. It is a very challenging yet quite approachable science. There are great model systems (arguably the best in the biopharma industry), and the probability of successful drug development is among the highest across all therapeutic areas (1). Those in the AMR field contribute to a wonderful, supportive community. The subject matter impacts so many areas of medicine, the efforts truly have global reach, and the impact on patient and caregiver lives is tremendous.

However, I was mentally and emotionally exhausted from “fighting the fight,” trying to justify our team’s existence, trying to come up with commercial models that could achieve the rising expectations of big pharma and their investors. I needed to feed my family. I was tired of being a victim of our own successes (“Thank you, but no thank you”). Investors were not interested in the area, or their interests were misaligned with what was clinically meaningful. The new talent and existing leadership pool grew smaller and smaller. Then there were the politics. Literally. So, I chose to leave the field as my full-time career and have remained an active community participant while also cheering from the sidelines for those brave enough to remain fully committed.

During the time in which I stepped away from anti-bacterial R&D, I contributed to a study of the talent drain out of the anti-bacterial space and helped to spearhead an effort to re-invigorate a rigorous and focused community of anti-bacterial and AMR experts, ranging from those in fundamental research to those in clinical practice. I participated in the launch and subsequent wind-downs of infectious disease-focused start-ups, led multiple in- and out-licensing deals around anti-infectives, and did diligence on dozens of other anti-microbial assets and technologies. I spoke frankly and openly with investors and experts in the area about their own experiences and what it would take to overcome the systemic challenges. From my many years of hands-on anti-bacterial R&D experience, from the learnings I gained from conversations with many of my generous peers, and more recently from my vantage point as an investor, board member, and dealmaker, I am able to summarize my findings and recommendations in this Perspective.

First, the data clearly demonstrate that the number of AMR researchers has significantly declined over the past 20 years, the AMR research output is orders of magnitude lower than in fields like oncology, and the current AMR R&D workforce is limited and declining, as are the number of investors and investor dollars into the space, even as the AMR threat grows (https://www.amrindustryalliance.org/wp-content/uploads/2023/02/Leaving-the-Lab_final-1.pdf). At the same time, the number of newly minted physicians choosing ID as a specialty is also declining, as residency classes fail to fill their seats (2).

In addition to fewer trial investigators, pharma consultants and advisors, manuscript reviewers, study section members, fewer experts to advise or become policy makers, and fewer experts to communicate to the public, the collateral damage from this talent drain ultimately leads to critically few experts to train the next generation. Newcomers will make the same mistakes and suffer from a lack of constructive criticism and mentoring, as they will have difficulty finding a strong anti-bacterial community with the appropriate talent and resources to guide them. With fewer investors in the space, there are diminishing financial incentives for talent to stay, as there are considerably lower chances of big upsides compared with investments in other more lucrative therapeutic areas.

So, why do experts stay in the space, despite these challenges, and how do we reverse the talent drain trend? What I heard, first and foremost, and what drives me to stay engaged as much as I do is that the talent that enters and remains in ID is mission driven. They find the space rewarding and compelling. They have a personal passion for public health and the quest for radical cures for diseases that plague so many diverse segments of the population. In terms of identifying and developing new talent, seasoned leaders see the mutual benefit to teaching and mentoring, to working side-by-side with others with a shared passion, to helping new talent to see the more-than-incremental gain that can be achieved from their work, and how they can contribute to a real change in the practice of medicine. My conversations with senior talent in the ID space highlighted the importance of sharing science within and outside their own organizations, encouraging presentations at journal clubs, publishing their work, sharing their challenges and achievements at meetings and conferences, and, whenever possible, encouraging field-based learning. Our discussions also highlighted the need to expose talent to non-traditional career paths, such as within local, state, and federal government, careers in regulatory sciences, and careers in scientific communications, for example. Fortunately, the American Society for Microbiology and the Infectious Diseases Society of America recently announced the launch of a new meeting, in 2025, that intends to provide a venue for the end-to-end AMR community to gather and share updates and which will hopefully lead to continued sharing opportunities (https://asm.org/press-releases/2024/december/asm-and-idsa-to-launch-new-conference-on-amr). Moreover, my discussions emphasized the need to always say “yes” to mentoring and, just when you thought you could not, mentor more.

From an incentive and investment perspective, I believe and have heard, loud and clear, that what is needed is a radical departure from the status quo. Some see transferable vouchers or tax credits as tangible incentives with real value that will have meaningful impact on investor and partnership decisions (https://eur-lex.europa.eu/legal-content/EN/TXT/PDF/?uri=CELEX:52023DC0190; https://ec.europa.eu/commission/presscorner/api/files/attachment/875387/Factsheet%20Pharma%20AMR_EN.pdf). Many also see a need to carve anti-bacterials and AMR diagnostics out of the existing diagnosis-related group classification model (https://www.cms.gov/icd10m/version38-fullcode-cms/fullcode_cms/Design_and_development_of_the_Diagnosis_Related_Group_(DRGs).pdf). Several peers that I spoke with agreed with my own perspective that (fortunately) AMR infections, specifically with multidrug-resistant and extensively drug-resistant pathogens, still constitute a rare disease situation and that this needs to be reflected in the pricing/reimbursement models (https://www.fda.gov/media/99546/download). My discussions importantly pointed to a need to communicate the unrecognized sources of value of anti-bacterials with very clear messaging, akin to what is demonstrated clinically and reflected in the pricing and reimbursement of HepC, cardiovascular, and of course GLP-1 drugs (https://healthpolicy.usc.edu/article/generalized-risk-adjusted-cost-effectiveness/). Investors and politicians need to engage in this re-focusing of the value conversation, as they have the ear of many components of the critical audience. Moreover, all agree that non-dilutive financial supporters are essential to the space. Many traditional investors want to see validation from the non-dilutive backers before they will jump in. Increased engagement from investors not only supports the financing of programs but also incentivizes experienced talent to move into and stay in the anti-infective space when they have many other more lucrative therapeutic area options to choose from. Moreover, it goes without saying that we need to incentivize physicians to enter the ID specialty. Increasing pay, lowering loan burdens, and supporting better working conditions and respect that they are due as valuable contributors to patient and organizational health, especially from the more traditionally “prestigious” clinical and corporate colleagues, will go a long way.

Finally, we need to empower new and existing talent to shift the public perception about infectious diseases and AMR!

- “The hospital is dirty/the surgeon made mistakes.”
- “Too many Rx being written/inappropriate use.”
- “The patient had unsafe practices.”

These narratives create the perception that infection is “dirty” and “preventable” and that if you “just change behavior,” this whole AMR problem goes away. As we know, the situation is far more complex, and for many patients, infection is likely inevitable, recurring, and ultimately untreatable due to no fault of their own or of their physicians or caregivers. Appropriate use of antibiotics also results in resistance. Antibiotic resistance is an outcome of basic biology.

Here’s the bottom line: to reverse the talent drain and to discover, develop, and commercialize game-changing anti-bacterials, we need to stop dabbling, shift the messaging, shift the economics, and start investing. Big pharma needs anti-microbials to sustain the development and clinical success of their strategically important therapeutics (3). Oncology, neurosurgery, gastroenterology, respiratory, immunology, and transplantation medicine can all place clinical trial subjects and patients on approved medications, at risk of infection. Furthermore, this risk may increase as we move to more complex therapies and more vulnerable patient populations. Large and small companies that “dabble” in the space waste precious resources, delay progress, demoralize employees, and contribute to false narratives around solutions to the challenges. As a 15 year resident of the SF Bay Area who has watched “solutions” for the unhoused population fail over and over, I can see some parallels to the various attempted fixes to the AMR situation. You can patch holes and pat yourselves on the back, but the reality is that the patches can be distractions, if not true dangers, and we cannot afford to kick the can down the road for another 10 years. Let us fix the market, fix the investment theses, and fix the talent drain. The science is hard enough. We need considerable lead time to re-build the industry. Let us hope that there is no major outbreak of AMR infection in the interim!
